# Highly Specific PET Imaging of Prostate Tumors in Mice with an Iodine-124-Labeled Antibody Fragment That Targets Phosphatidylserine

**DOI:** 10.1371/journal.pone.0084864

**Published:** 2013-12-19

**Authors:** Jason H. Stafford, Guiyang Hao, Anne M. Best, Xiankai Sun, Philip E. Thorpe

**Affiliations:** 1 Department of Pharmacology, The Simmons Cancer Center, University of Texas Southwestern Medical Center, Dallas, Texas, United States of America; 2 Department of Radiology, The Advanced Imaging Research Center, University of Texas Southwestern Medical Center, Dallas, Texas, United States of America; Genentech, United States of America

## Abstract

Phosphatidylserine (PS) is an attractive target for imaging agents that identify tumors and assess their response to therapy. PS is absent from the surface of most cell types, but becomes exposed on tumor cells and tumor vasculature in response to oxidative stresses in the tumor microenvironment and increases in response to therapy. To image exposed PS, we used a fully human PS-targeting antibody fragment, PGN635 F(ab’)_2,_ that binds to complexes of PS and β2-glycoprotein I. PGN635 F(ab’)_2_ was labeled with the positron-emitting isotope iodine-124 (^124^I) and the resulting probe was injected into nude mice bearing subcutaneous or orthotopic human PC3 prostate tumors. Biodistribution studies showed that ^124^I-PGN635 F(ab’)_2_ localized with remarkable specificity to the tumors with little uptake in other organs, including the liver and kidneys. Clear delineation of the tumors was achieved by PET 48 hours after injection. Radiation of the tumors with 15 Gy or systemic treatment of the mice with 10 mg/kg docetaxel increased localization in the tumors. Tumor-to-normal (T/N) ratios were inversely correlated with tumor growth measured over 28 days. These data indicate that ^124^I-PGN635 F(ab’)_2_ is a promising new imaging agent for predicting tumor response to therapy.

## Introduction

Phosphatidylserine (PS) is an attractive target for cancer imaging agents that can be used for disease diagnosis, staging and therapeutic planning. PS is a phospholipid that is generally not found on the surface of normal cells because lipid-specific transporters sequester it in the inner leaflet of the cell’s plasma membrane [1,2]. When cells undergo apoptosis, as do tumor cells responding to chemotherapy, PS becomes exposed on their outer membrane surface through one or more calcium-dependent mechanisms [3,4]. PS exposure is also induced on the viable vascular endothelium in tumors by oxidative stresses within the tumor microenvironment [5-7] and increased PS exposure levels on the endothelium are consistently seen in tumors responding to therapy [8-11]. Since PS exposure on tumor endothelium and tumor cells correlates with tumor growth inhibition [8,9,12], it provides an excellent marker for predicting tumor response to therapy. 

Several PS-targeting strategies have been employed to image tumors and determine their response to therapy. The PS binding protein, annexin V, has been radiolabeled with various positron emitters for positron emission tomography (PET) of tumors in several animal models [13-15]. Technetium-99m (^99m^Tc)-labeled annexin V has been used for single photon emission computed tomography (SPECT) in humans and has shown prognostic value for head and neck cancer, late stage lung cancer and lymphoma [16,17]. Others have used the C2A domain of radiolabeled synaptotagmin I for PET and SPECT imaging of lung carcinomas in animals treated with paclitaxel [18,19]. Low molecular weight PS imaging probes, such as dipicolylamine-Zn^2+^ complexes [20], are also in development. While these probes have demonstrated diagnostic value, they all display unfavorable biodistributions with high abdominal background signal due to probe accumulation in the liver and kidneys. 

We have developed a family of PS-targeting monoclonal antibodies that reactivate tumor immunity and induce immune cell-mediated destruction of tumor vasculature. *In vivo* screening methods were designed to identify antibodies that bound PS directly, but further characterization of the antibodies revealed that they interact with PS by forming high affinity complexes with the serum protein β2-glycoprotein I (β2GP1) [21]. The family of antibodies is named after a human-mouse chimeric antibody known as bavituximab that is currently being evaluated in clinical trials in cancer patients as an adjuvant to chemotherapy. Unlike PS-targeting antibodies that cause antiphospholipid syndrome (APS), bavituximab does not promote thrombosis and is well-tolerated in patients in doses as high as 4 mg/kg. 

Bavituximab has higher specificity for PS than does annexin V and higher affinity than many lower molecular weight molecules known to bind PS [21]. These characteristics suggest that bavituximab and similar PS-targeting antibodies may not only be useful for cancer therapy, but that they may also be useful for cancer imaging. We have previously shown that bavituximab labeled with the ^74^As (t_1/2_ = 17.8 days) gave clear PET images of subcutaneous prostate tumors in rats [22]. Optimal images were obtained 72 h after injection, when concentrations of the probe in the blood had fallen to levels that did not obscure signal from the tumor [22]. 

The most recent addition to the bavituximab family is a fully human PS-targeting antibody named PGN635. PGN635 (K_d_ = 1.8 nM) binds with similar affinity as bavituximab but, because it lacks mouse protein sequences, has a higher potential for clinical translation. To obtain shorter blood residence times than those required for ^74^As-bavituximab imaging, we used the F(ab’)_2_ fragment of PGN635 instead of the intact antibody. Iodine-124 (^124^I) was chosen to label the antibody fragment since its radioactive half-life (t_1/2_ = 4.2 days) has been shown to be compatible with immuno-PET and it has been increasingly studied in clinic [23,24]. Moreover, ^124^I allows direct labeling of the antibody fragment by electrophilic radioiodination whereas other PET isotopes commonly used for immuno-PET such as copper-64 (^64^Cu) and zirconium-89 (^89^Zr) require chelator/linker molecules [25]. 

Here we report the ability of ^124^I-lableled PGN635 F(ab’)_2_ to image prostate tumors growing in mice by PET. ^124^I-PGN635 F(ab’)_2_ produced clear PET images of subcutaneous and orthotopic prostate tumors in mice. Treatment with chemotherapy or radiotherapy increased tumor uptake of ^124^I-PGN635 F(ab’)_2_ and the tumor to normal tissue (T/N) ratios correlated with the subsequent tumor growth inhibition. These data suggest that ^124^I-PGN635 F(ab’)_2_ could be used as a diagnostic tumor imaging agent and for predicting tumor response to therapy. 

## Materials and Methods

### Radiochemistry

Iodine-124 (^124^I) was purchased from IBA Molecular, Inc. (Richmond, VA) and Iodine-125 (^125^I) was purchased from Perkin Elmer (Waltham, MA). Iodination tubes and Protein A agarose were from Pierce Biotechnology (Rockford, IL). Instant thin-layer chromatography plates (ITLC-SG) were from Pall Life Sciences (East Hills, NY). Horse-radish peroxidase (HRP) conjugated streptavidin was purchased from Jackson Immunoresearch Labs (West Grove, PA). Bio-Spin 6 gel filtration columns were purchased from Bio-Rad Laboratories (Hercules, CA). Recombinant human β2GP1 was obtained from Peregrine Pharmaceuticals, Inc. (Tustin, CA). 96-well Immulon 1B microtiter plates were purchased from Thermo LabSystems (Franklin, MA). L-α-phosphatidylserine (PS) was purchased from Avanti Polar Lipids (Alabaster, AL). Furosemide was purchased from Sigma-Aldrich (St. Louis, MO). Docetaxel was obtained from the UT Southwestern pharmacy (Dallas, TX). 

The fully human PS-targeting antibody, PGN635, was generated by Affitech A.S. (Oslo, Norway) in collaboration with Peregrine Pharmaceuticals, Inc. (Tustin, CA). For *in vitro* assays, PGN635 was mixed with an equal weight of human β2GP1 to enable binding to PS. Aurexis is a human monoclonal antibody that binds an irrelevant antigen (*S. aureus* clumping factor A) and was used as a negative control. PGN635 and Aurexis were produced under Good Manufacturing Practice (GMP) conditions. Goat anti-β2GP1 polyclonal antibody was purchased from Pierce Biotechnology (Rockford, IL). Horseradish peroxidase (HRP) conjugated donkey anti-goat IgG secondary antibody was purchased from Jackson Immunoresearch Labs (West Grove, PA). 

F(ab’)_2_ fragments were generated by reacting antibodies with pepsin (pH 4.0) at a molar ratio of 1:130 (antibody:pepsin) for 1 h at 37°C. F(ab’)_2_ fragments (MW = 110 kD) were purified on a FPLC S*-*200 column (Pharmacia, Piscataway, NJ). The F(ab’)_2_ fragments were then radioiodinated using the indirect IODO-GEN method (Pierce Biotechnology). Since radioiodination with ^124^I or ^125^I is considered to have equivalent effects on antibody binding [26,27], ^125^I was used for some experiments to minimize exposure to beta emissions. Briefly, 1-3 mCi of iodine were activated in 100 µl iodination buffer (125 mM Tris-HCL, pH 6.8, 150 mM NaCl) in a pre-coated iodination tube and then reacted with 0.2-0.6 mg F(ab’)_2_ in 100 µl iodination buffer in a separate uncoated tube on ice. Free iodine was removed with Bio-Spin 6 gel filtration columns that were pre-blocked with iodination buffer containing 10% FBS. Radio-TLC analysis was used to determine iodination efficiency on a Rita Star Radioisotope TLC Analyzer (Straubenhardt, Germany) using ITLC-SG plates. 

Human prostate carcinoma (PC3) cells were obtained from ATCC (Rockville, MD) and transfected to stably express firefly luciferase [28]. Adult bovine aortic endothelial (ABAE) cells were obtained from Clonetics (Walkerville, MD). PC3-luc cells were maintained in F-12K media, and ABAE cells were maintained in DMEM media. Tissue culture media was purchased from HyClone (Thermo Scientific, Logan, UT) and was supplemented with 10% FBS and 2 mM L-glutamine. 

### Tumor Models

For subcutaneous (s.c.) tumors, 2 x 10^6^ PC3-luc cells in matrigel/PBS (1:1) were injected into the upper right flank of male athymic nu/nu mice (Charles River, Frederick, MD). Tumor growth was monitored by measuring two perpendicular diameters and calculating tumor volume using the formula π/6 x D x d^2^ where D is the larger diameter and d is the smaller diameter. 

For orthotopic tumors, mice were anesthetized and a lower midline abdominal incision was made to expose the prostate capsule. The prostate capsule was opened and 10^6^ PC3-luc cells in 50 µl matrigel/PBS (1:1) were injected into the dorsal prostate. The internal membrane was then sutured and the skin was clipped to close the incision. Tumor growth was monitored by bioluminescence imaging (BLI) with an IVIS Lumina imaging system (Xenogen, Alameda, CA).

This study was approved by the Institutional Animal Care and Use Committee at UT Southwestern Medical Center (protocol#: 2009-0152). The experiments were carried out in strict accordance with NIH guidelines including making all efforts to minimize animal suffering. 

### *In Vitro* Binding Assays

To determine antibody binding to PS immobilized on plastic, 50 µl of PS in *n*-hexane (10 µg/ml) was added to wells of Immulon 1B 96-well plates. The solvent was evaporated at room temperature (RT) and the plates were blocked for 1 h with 1% BSA in PBS and then washed with PBS. ^125^I-PGN635 F(ab’)_2_ and unlabeled PGN636 F(ab’)_2_ were diluted in blocking buffer at an initial concentration of 200 µg/ml and 2-fold dilutions were performed in a separate 96-well plates (100 µl per well). Biotinylated PGN635 (0.1 µg/ml) and β2GP1 (1 µg/ml) were then added to each well (final volume/well = 200 µl). The mixtures (100 µl/well) were transferred from the dilution plates to PS-coated plates. The plates were incubated for 1 h at RT. The plates were washed and bound PGN635-biotin was detected with HRP-conjugated streptavidin (1:2000 in blocking buffer) and developed with the chromogenic substrate ODP. The plates were read at 490 nm with a microplate reader (BioTek Instruments, Winooski, VT). 

To assess antibody binding to cells, ABAE and PC3-luc cells were cultured in 12-well tissue culture plates (BD Falcon, Bedford, MA) until ~80% confluent. To induce PS exposure, cells were irradiated with 5 Gy. Non-irradiated cells were used as controls. After 24 h, the cells were incubated with 1 µg/ml ^124^I-PGN635 F(ab’)_2_ or ^124^I-Aurexis F(ab’)_2_ (negative control) for 1 h. The cells were then washed with PBS and dissolved with 1N NaOH (30 min, RT). Activity from cell digests was measured using a γ-counter (Perkin Elmer, Waltham, MA).

### *In Vivo* Biodistribution Studies

Mice bearing s.c. PC3-luc tumors were injected i.v. with 50 µg/1.85 MBq of ^125^I-PGN635 F(ab’)_2_. Animals were sacrificed 24 or 48 h after injection and tumors, blood, and other organs of interest were collected, weighed, and their radioactivity measured. ^125^I-PGN635 F(ab’)_2_ uptake in each organ was expressed as the percentage injected dose per gram tissue (% ID/g) and percentage injected dose per organ (% ID/organ) (n = 3). 

### PET Imaging of Tumors with ^124^I-PGN635 F(ab’)_2_

Mice bearing s.c. or orthotopic PC3-luc tumors were selected for imaging with a Siemens Inveon PET-CT Multimodality System (Siemens Medical Solutions Inc., Knoxville, TN). Thyroid uptake of ^124^I was blocked by adding 10 drops saturated KI per 100 ml of drinking water 24 h before injection of ^124^I-PGN635 F(ab’)_2_. Stomach uptake was blocked by gastric lavage with 1.5 ml potassium perchlorate in 200 µl PBS 30 min before injection. The mice were injected into the tail vein with 50 µg/1.85 MBq of ^124^I-PGN635 F(ab’)_2_ in 150 µl PBS. The mice were injected i.p. with 10 mg/kg furosemide and given 200 µl water by gastric lavage 1 h before imaging to clear residual activity from the bladder. For imaging, the mice were anesthetized with 3% isoflurane until stable vital signs were established and then placed on the imaging bed under 2% isoflurane for the duration of the procedure. CT images were acquired at 80 kV and 500 µA. CT images were reconstructed using COBRA Reconstruction Software. PET imaging was performed directly after acquisition of CT data, using the standard energy window of 350-650 keV. The scan time for PET images was between 10 and 20 min. The data were reconstructed using and the Fourier Rebinning and Ordered Subsets Expectation Maximization 3D (OSEM3D) algorithm provided by the Siemens Inveon Research Workplace (IRW) software. Reconstructed CT and PET images were also superimposed and analyzed using the IRW software. For quantification, tumor-margins were determined by CT morphology and regions of interest (ROIs) were defined manually. 

### Assessing Tumor Response to Therapy with ^124^I-PGN635 F(ab’)_2_

Treatment (n=3) was initiated in mice bearing subcutaneous PC3-luc tumors when the tumor volumes reached 0.3-0.7 cm^3^. Mice treated with chemotherapy (CTx) were injected i.p. with docetaxel (10 mg/kg). Mice treated with x-irradiation (xRT) received a single dose of 15 Gy to their tumors delivered with an XRAD 320 biological irradiator (Precision X-Ray, North Branford, CT). PET imaging of tumors with ^124^I-PGN635 F(ab’)_2_ was performed 24 h later. Tumor/normal (T/N) ratios were calculated as the % ID/g tumor/% ID/g muscle (left forelimb) at 48 h after injection of ^124^I-PGN635 F(ab’)_2_. Tumor growth was monitored for 28 days after imaging. The tumor volume immediately before treatment (Vol_0_) and 28 days after treatment (Vol_28_) were recorded for each individual animal. The tumor growth index for each animal was calculated as Vol_28_/Vol_0_. The correlation coefficient (Pearson’s r) between the T/N ratio and the tumor growth index was calculated using log values. 

## Results

### ^124^I-PGN635 F(ab’)_2_ Binding and Stability

**Figure 1:**
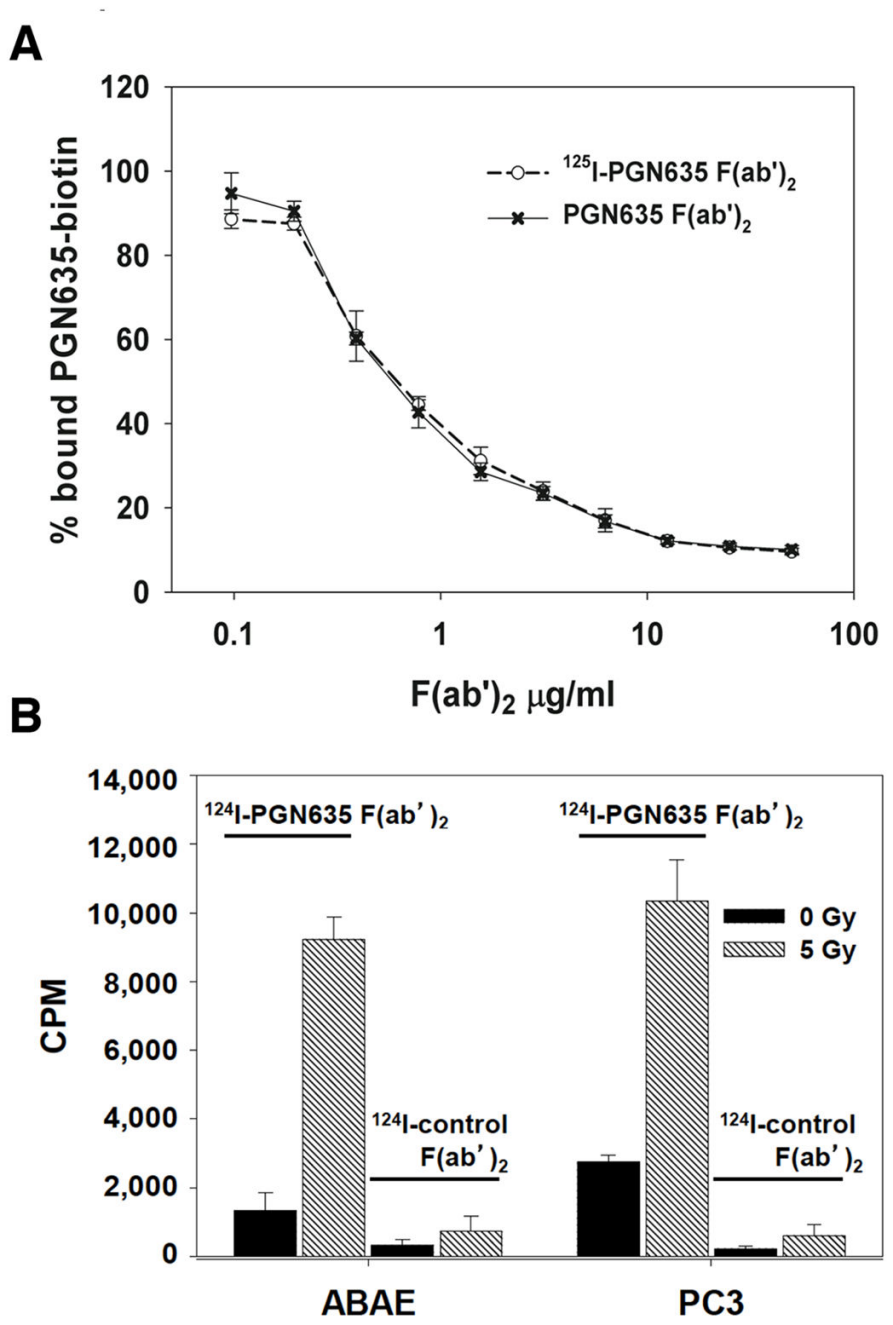
Radioiodination does not affect binding of PGN635 F(ab’)_2_ to PS. **A**) Competition ELISA shows ^125^I- PGN635 F(ab’)_2_ and unlabeled PGN635 F(ab’)_2_ have similar ability to compete with PGN635-biotin for binding to immobilized PS. **B**) Binding of ^124^I-PGN635 F(ab’)_2_ to PS-expressing cells. PS-exposure on ABAE and PC3-luc cells was induced by irradiation with 5 Gy 24 h before the binding assay. Non-irradiated cells served as controls. The cells were incubated with ^124^I-PGN635 F(ab’)_2_ or ^124^I-control F(ab’)_2_ for 1 h. The cells were then washed and dissolved in NaOH (1N) and ^124^I was quantified by gamma counting (CPM = counts/min.). Points and histograms, means; bars, + S.D.

 PGN635 F(ab’)_2_ was radioiodinated with labeling efficiencies ranging between 14.1% and 19.5%. Radioiodination did not affect the ability of the antibody fragment to bind PS immobilized on plastic. Figure **1A** shows that a 10-fold excess of ^125^I-PGN635 F(ab’)_2_ or unlabeled F(ab’)_2_ inhibited the binding of PGN635-biotin to PS by 55.5% and 57.3%, respectively. Radioiodination also did not affect the ability of PGN635 F(ab’)_2_ to bind PS exposed on the surface of irradiated endothelial cells or irradiated prostate tumor cells (Figure **1B**). 

 To determine the stability of ^124^I-PGN635 F(ab’)_2_ *in vivo*, serum collected from mice injected with ^124^I-PGN635 F(ab’)_2_ was analyzed by HPLC size exclusion chromatography. There was no evidence of lower molecular weight ^124^I-labeled degradation products or free ^124^I in the circulation 48 h after injection of the probe (Figure **S1A**). The majority of the ^124^I-PGN635 F(ab’)_2_ eluted earlier than the control ^124^I-PGN635 F(ab’)_2_ suggesting that the antibody had bound to a plasma protein. To determine if the increased molecular weight of the ^124^I-PGN635 F(ab’)_2_ was due to binding to mouse β2GP1 *in vivo*, mice were injected with full-length PGN635 and 24 h later the antibody was recovered from serum with protein A agarose. Western blotting with antibodies to β2GP1 revealed that the PGN635 co-purified with mouse β2GP1 (Figure **S1B**). Mouse β2GP1 is more highly glycosylated than human β2GP1 [29] and therefore, migrated slightly slower during gel electrophoresis.

### Biodistribution of ^125^I-PGN635 F(ab’)_2_

**Figure 2:**
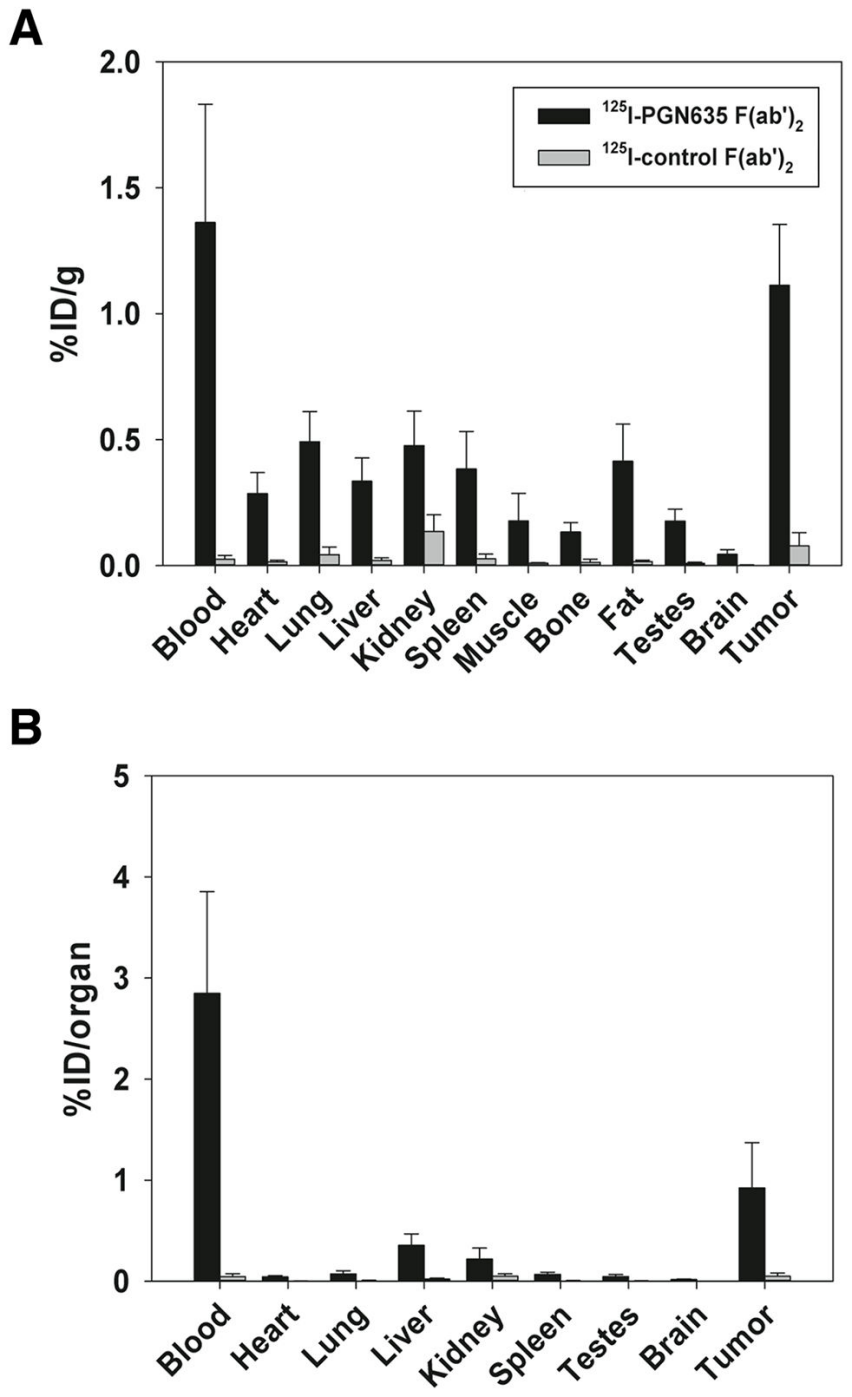
^125^I-PGN635 F(ab’)_2_ biodistribution. Mice (n =3) bearing s.c. PC3-luc tumors were injected with 1.85 MBq (50 µg) of ^125^I-PGN635 F(ab’)_2_ or ^125^I-control F(ab’)_2_. Antibody distribution to the indicated organs was determined after 48 h by counting the radioactivity with a gamma counter. **A**) Biodistribution by percent injected dose per gram (%ID/g) of tissue. **B**) Biodistribution by percent injected dose per organ (%ID/organ). The % ID in the blood was calculated assuming a blood volume of 2.18ml/25 g body weight.

Biodistribution studies were conducted in male nu/nu athymic mice bearing subcutaneous PC3 tumors to evaluate uptake of ^125^I-PGN635 F(ab’)_2_ in specific tissues. 24 h after i.v. administration of ^125^I-PGN635 F(ab’)_2_ activity in the blood was 6.7% ID/g (10.9% ID/organ) whereas uptake in all other tissues, including tumor, was <3% ID/g (<2% ID/organ) (Figure **S2**). ^125^I-Aurexis F(ab’)_2_ (control) displayed low uptake in all tissues with an activity in the blood of only 0.2% ID/g (0.4% ID/organ) after 24 h. After 48 h, ^125^I-PGN635 F(ab’)_2_ was predominantly localized to the tumor (1.2 % ID/g) and blood (1.4 % ID/g) (Figure **2**). The tumor:blood ratio was 0.9:1 whereas the tumor:liver ratio was 3.3:1. 

### MicroPET Imaging of PC3-luc Tumors with ^124^I-PGN635 F(ab’)_2_

**Figure 3:**
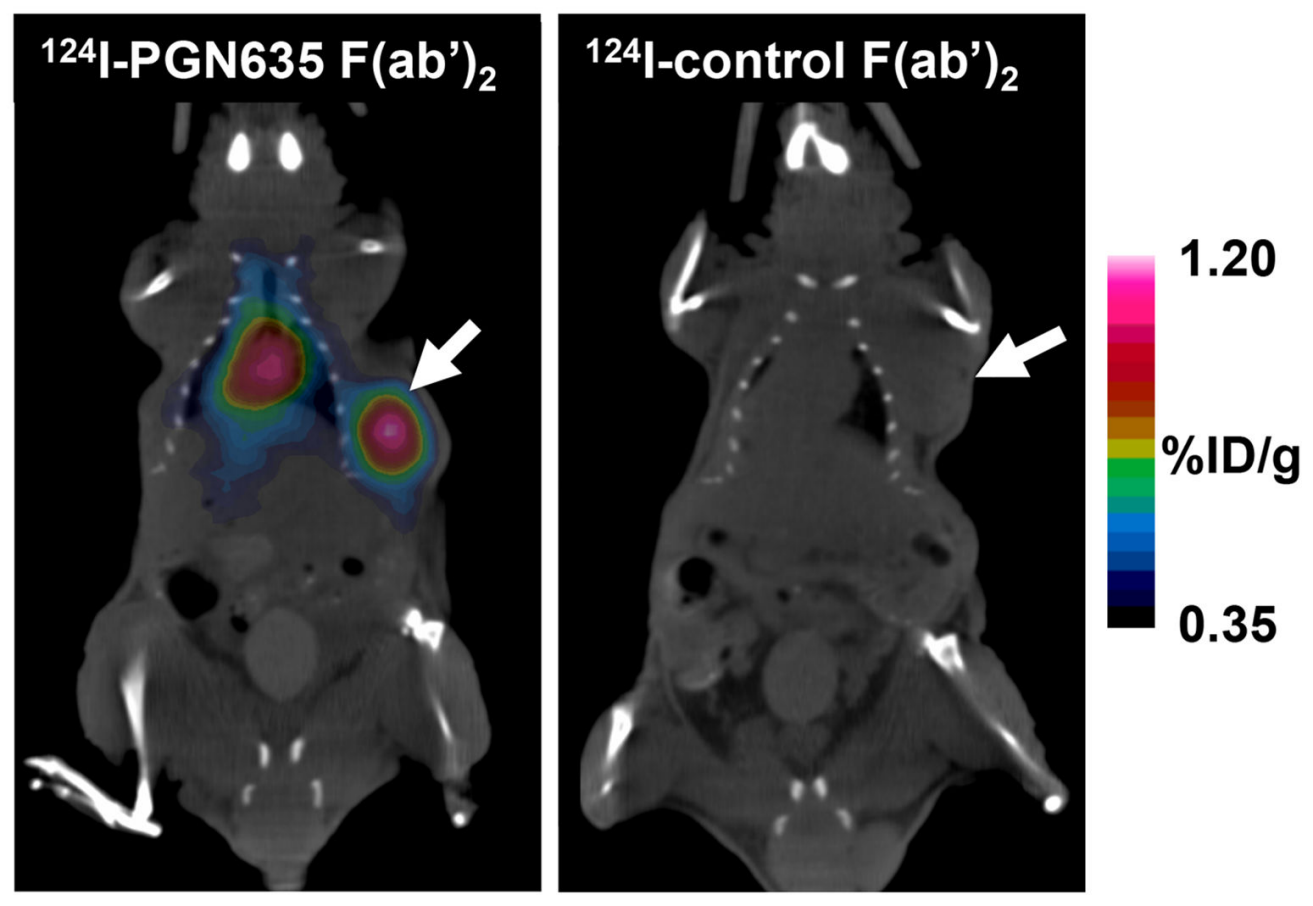
PET imaging of subcutaneous PC3 tumors with ^124^I-PGN635 F(ab’)_2_. Mice bearing s.c. PC3-luc tumors were injected with ^124^I-PGN635 F(ab’)_2_ or ^124^I-control F(ab’)_2_. The animals were imaged by PET/CT 48 h later. Reconstructed PET and CT images were fused and analyzed. The images clearly show preferential labeling of the tumor by PET (arrow). Signal from the heart is due to ^124^I-PGN635 F(ab’)_2_ in the blood. ^124^I-control F(ab’)_2_ did not label tumors and was rapidly cleared from the circulation. Representative mice from groups of 4 mice are shown.

Mice bearing subcutaneous PC3-luc tumors growing in their right flank were injected i.v. with ^124^I-PGN635 F(ab’)_2_ and imaged at 24 and 48 h. Tumor-bearing mice injected i.v. with ^124^I-Aurexis F(ab’)_2_ were used as negative controls. After 24 h, the high background signal from normal tissues did not allow for specific imaging of the tumor. However, 48 h after injection ^124^I-PGN635 F(ab’)_2_ uptake in the tumor was significantly higher than background allowing clear delineation of the tumor (Figure **3**, Figure **S3**). Average tumor uptake of ^124^I-PGN635 F(ab’)_2_ determined by PET signal quantitation was 1.2% ID/g. Whereas average uptake of the probe in the heart, liver and muscle was 0.9, 0.7, and 0.2 %ID/g respectively. ^124^I-Aurexis F(ab’)_2_ (control) did not show significant tumor uptake (0.03 %ID/g).

**Figure 4:**
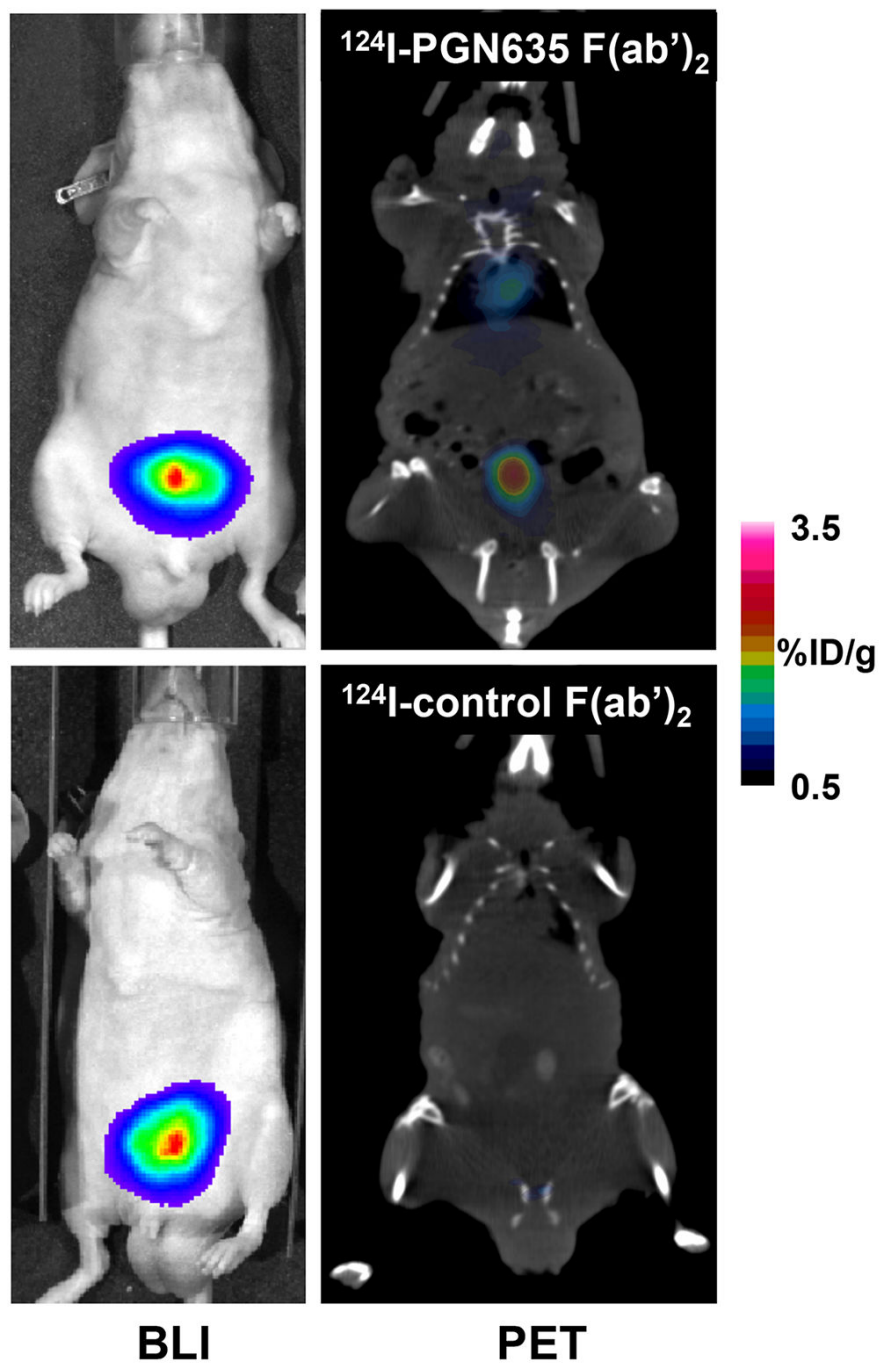
PET imaging of orthotopic PC3-luc tumors with ^124^I-PGN635 F(ab’)_2_. Mice bearing orthotopic PC3-luc tumors were injected with ^124^I-PGN635 F(ab’)_2_ or ^124^I-control F(ab’)_2_. PET and BLI imaging were performed 48 h later. The PET images clearly show preferential labeling of the orthotopic prostate tumor. The PET images of the tumors were coincident with the BLI images. ^124^I-control F(ab’)_2_ did not label the tumors. Representative mice from groups of 4 mice are shown.

^124^I-PGN635 F(ab’)_2_ also imaged orthotopically-implanted PC3-luc tumors (Figure **4**, Figure **S4**). Average uptake of ^124^I-PGN635 F(ab’)_2_ in the prostate tumors 48 h after injection was 1.6% ID/g. Localization of the probe to the prostate was confirmed by coincidental bioluminescence imaging. Tumor size and localization of ^124^I-PGN635 F(ab’)_2_ correlated to the size and location of the BLI signal. ^124^I-Aurexis F(ab’)_2_ (control) did not localize to orthotopic tumors (0.08 %ID/g). 

### Assessing Tumor Response to Therapy with ^124^I-PGN635 F(ab’)_2_

**Figure 5:**
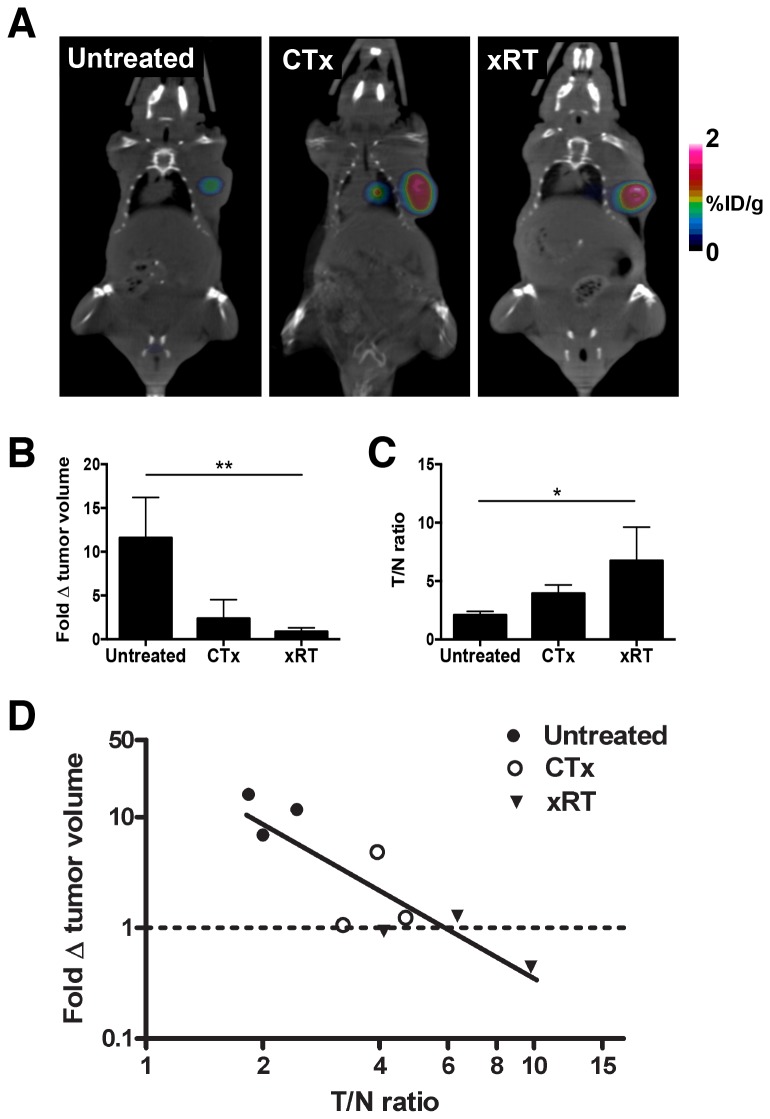
^124^I-PGN635 F(ab’)_2_ localization predicts tumor response to therapy. **A**) Mice bearing PC3-luc tumors were treated with 10 mg/kg docetaxel (CTx) or their tumors were irradiated with 15 Gy (RTx). After 24 h, the mice were injected with ^124^I-PGN635 F(ab’)_2_ and imaged by PET 48 h later. **B**) CTx and RTx significantly inhibited tumor growth (one-way ANOVA, P < 0.01). **C**) ^124^I-PGN635 F(ab’)_2_ uptake was significantly higher in treated tumors (one-way ANOVA, P < 0.05). **D**) The tumor-to-normal (T/N) ratio inversely correlated with the tumor growth over the next 28 days (Pearson’s r = -0.85, P < 0.01). The tumor volume on day 28 was divided by the volume on the day of treatment to calculate the fold change in tumor volume. The T/N ratio at the time of imaging was predictive of the tumor response.

Since chemo- and radiotherapy increase the exposure of PS on apoptotic tumor cells [30,31] and the tumor vasculature [8-10], we determined whether therapy would increase the localization of ^124^I-PGN635 F(ab’)_2_ . Mice bearing PC3-luc tumors were treated with 10 mg/kg docetaxel or with 15 Gy x-irradiation and were injected with ^124^I-PGN635 F(ab’)_2_ 24 h later. Forty-eight hours later, the mice were imaged by PET. Figure **5A** shows that while the tumors in all groups were similar sizes at the time of imaging, both treatments increased tumor localization of ^124^I-PGN635 F(ab’)_2_. In untreated control mice the average tumor to normal tissue (muscle) ratio (T/N) was 2.1 (Figure **5C**). Chemotherapy (CTx) increased the T/N ratios to an average of 3.9, while radiotherapy (RTx) increased the T/N ratios to an average of 6.7 (Figure **5C**). Figure **5D** shows that the T/N ratios were inversely correlated with the subsequent tumor growth measured over 28 days (Pearson’s r = -0.85; P<0.01). Untreated tumors increased in volume by an average of 11.5-fold whereas the volume of tumors treated with CTx increased on average by only 2.4-fold (Figure **5B**, Figure **S5**). RTx was more effective at inhibiting tumor growth than CTx with the volume of irradiated tumors decreasing on average by 10% (Figure **5B**, Figure **S5**). These data indicate that imaging with ^124^I-PGN635 F(ab’)_2_ may be useful for predicting tumor response to therapy. 

## Discussion

 The present study demonstrates that the ^124^I-labeled F(ab’)_2_ fragment of the fully human PS-binding antibody PGN635 provides clear PET images of subcutaneously and orthtopically-implanted PC3 prostate tumors in mice. Tumor localization also correlated with inhibition of tumor growth in animals treated with docetaxel or irradiation suggesting that ^124^I-PGN635 F(ab’)_2_ could be useful for predicting tumor response to therapy in patients. In addition to its *in vivo* activity, the radioiodinated antibody fragment fully retained its structural integrity *in vivo* and its ability to bind to immobilized PS and to PS-expressing irradiated cells *in vitro*.

 We previously labeled the full-length chimeric IgG bavituximab with ^74^As and obtained clear images of solid tumors in rats by PET [22]. Recently, Ogasawara et al. demonstrated that full-length PGN635 labeled with ^89^Zr could image apoptotic tumors in mice [32]. In the present study, the F(ab’)_2_ fragment of PGN635 was used instead of full length IgG because its faster blood clearance should yield greater T/N ratios at earlier time points [33,34]. Preliminary imaging of tumor-bearing mice with F(ab’)_2_ fragments labeled with ^64^Cu showed high PET signals from the liver (16.3% ID/g) and kidneys (23.5 %ID/g) (unpublished data). We hypothesized that high uptake in these organs was due to transchelation of ^64^Cu from the 1,4,7,10-tetraazacyclododecane-1,4,7,10-tetraacetic acid (DOTA) chelator required for radiolabeling to copper-binding proteins such as Cu^2+^/Zn^2+^ super oxide dismutase (SOD1)[35]. Therefore, we chose to use iodine-124 as our reporter nuclide because it allowed us to directly label the PG635 F(ab’)_2_ without the use of a chelator/linker molecule. 

^124^I-PGN635 F(ab’)_2_ did indeed allow for more rapid imaging of tumors than ^74^As-bavituximab [22], but the blood half-life was longer than is typical of a F(ab’)_2_ fragment. We have previously reported that PGN635 F(ab’)_2_ labeled with a near-infrared dye (800CW-PGN635 F(ab’)_2_) was cleared from blood with a half-life of approximately 6 hours [36]. ^125^I-PGN635 F(ab’)_2_ was observed to have a similar half-life with 6.5% ID/g of remaining in the blood at 24 h, as compared to 0.2% ID /g for the control Aurexis F(ab’)_2_. The longer than expected blood half-life of ^125^I-PGN635 F(ab’)_2_ is most likely due to the generation of 210 kDa F(ab’)_2_/ β2GP1complexes that are cleared at a rate that is significantly slower than that of the 110 kDa Aurexis F(ab’)_2_ control. For this reason, optimal imaging with ^124^I-PGN635 F(ab’)_2_ was not obtained until approximately 48 h after injection when blood levels of the isotope had fallen to levels where they did not obscure antibody localization in the tumor. 

 The 48 hour lag time between ^124^I-PGN635 F(ab’)_2_ injection and tumor imaging along with the higher radiation burden associated with ^124^I decay may be identified as significant drawbacks when compared to fludeoxyglucose (^18^F-FDG), the current standard for tumor imaging by PET. ^18^F-FDG allows imaging within 2 hours of injection, rapidly decays (t_1/2_ = 110 min), and is excreted within 24 hours [37]. However, the biodistribution of ^124^I-PGN635 F(ab’)_2_ may be preferable to that of ^18^F-FDG for imaging some malignancies. ^18^F-FDG labels tumors because they take up and metabolize high amounts of glucose, but normal tissues also metabolize glucose. Thus, ^18^F-FDG imaging can suffer from relatively high background throughout the body and in particular, the brain and kidneys [38]. Furthermore, ^18^F-FDG is not effective for diagnosing prostate cancer since well-differentiated, androgen-dependent prostate carcinomas do not metabolize high amounts of glucose [39]. By comparison, the clarity of prostate tumor imaging with ^124^I-PGN635 F(ab’)_2_ was remarkable. At 48 h after injection there was no significant signal from normal tissue, with the exception of the heart because of the relatively large pooling of blood. 

 PET quantification with ^124^I can be difficult due its high energy positrons and complex decay scheme that results in single photon emission in the same energy window as the annihilation photons used for image reconstruction [40]. Various methods of data correction have been implemented to address these problems [41], but the background signal from ^124^I-PGN635 F(ab’)_2_ in normal tissues was low enough to allow us to use standard PET quantification protocols. Importantly, we observed no significant difference between the biodistribution of ^125^I-PGN635 F(ab’)_2_ and quantification of ^124^I-PGN635 F(ab’)_2_ PET in PC3-luc tumors at 48 h after injection. 

 In neither the present, nor the earlier study of 800CW-PGN635 F(ab’)_2_ [36], was there accumulation of antibody in the liver and kidneys. Uptake in these organs has been problematic for imaging with ^99^Tc-labeled annexin V and the C2 domain of synaptotagmin [18,42]. Different linkers such as hydrazinonicotinamide (HYNIC) and mercapoacetyltriglycine (MAG3) have been used to limit transchelation of ^99^Tc, but the resulting annexin-based probes exhibited only a modest improvement in biodistribution [43,44]. Localization of annexin V and synaptotagmin to the liver and kidneys is not precisely understood, but is probably due, at least in part, to nonspecific uptake systems in these organs that capture and metabolize low molecular weight proteins [45,46]. It is also possible that annexin V binds molecules other than PS in these organs as it has been shown to bind anionic polysaccharides such as heparan sulfate [47]. Finally, ^124^I-PGN635 F(ab’)_2_ PET did not label the skeletons of the mice compared to ^89^Zr-PGN635 which releases a small amount of free ^89^Zr that reacts non-specifically with mineral bone [32]. 

 The data showing a relationship between the level of tumor localization and therapeutic efficacy suggests that an important clinical use for ^124^I-PGN635 F(ab’)_2_ may be assessing tumor response to therapy. PS is constitutively expressed at low levels on several types of viable tumor cells [48], but large increases in PS exposure occur in response to therapy as a result of apoptotic and necrotic changes [49]. Prior work supports this possible clinical application. We previously demonstrated that PGN635 binds specifically to PS exposed on tumor cells and tumor endothelial cells and that binding is increased in tumors treated with radiotherapy [36]. Gong et al. showed that tumor localization of 800CW-PGN635 F(ab’)_2_ in mice was increased 4-fold 24 h after administration of docetaxel (unpublished data). Osagawara et al., showed that non-small cell lung cancer tumors exhibited more than a 6-fold increase in uptake of ^89^Zr-PGN635 in mice treated with an agonistic death receptor antibody that activates extrinsic apoptosis and a 2.5-fold increase after administration of paclitaxel that induces intrinsic apoptosis [32]. SPECT imaging studies with ^99m^Tc-labeled annexin V have demonstrated a correlation between apoptosis imaging and tumor response in cancer patients as early as 24 h after treatment initiation [17,50]. PET imaging studies with ^18^F-labeled annexin V have also shown a correlation between imaging and tumor response in mice [15]. Consistent with these prior reports, we found that prostate tumors in mice treated with chemotherapy or radiotherapy showed a 2.5 to 5-fold higher uptake of ^124^I-PGN635 F(ab’)_2_ relative to untreated tumor-bearing control mice. 

Although the T/N ratios in treated and untreated tumors were predictive of the subsequent tumor response, we found that total tumor uptake (%ID/g) of ^124^I-PGN635 F(ab’)_2_ was less than that reported for ^89^Zr-PGN635 [32]. The difference between the two probes may be attributed to the FcRn receptors on endothelium maintaining serum levels of the full-length IgG construct and allowing it more time to bind the wave of PS exposure induced by treatment. Alternatively, the difference may be due to the different tumor models and induction of apoptosis by the extrinsic versus intrinsic pathways. 

## Conclusion

Our findings indicate that ^124^I-PGN635 F(ab’)_2_ is a highly specific tumor imaging agent. Images obtained with this probe are substantially clearer than other PS-targeting probes because of very low uptake in normal tissues. The 48 h lag time between probe injection and tumor imaging may complicate clinical translation, but the data suggest ^124^I-PGN635 F(ab’)_2_ could still be a valuable tool for predicting how a tumor will respond to therapy. Ideally, the patient’s tumor would be imaged before initiation of therapy and again early in the course of treatment. Increases in tumor signal would be indicative of effective therapy. Conversely, lack of increase in tumor signal would indicate a change of treatment strategy could be required. Since exposed PS appears to be a universal marker of tumor vasculature and is universally exposed on tumors responding to therapy, ^124^I-PGN635 F(ab’)_2_ could have broad application for tumor detection and prediction of response to treatment in cancer patients. 

## Supporting Information

Figure S1 **^124^I-PGN635 F(ab’)_2_ is stable *invivo* and binds serum β2GP1.** **A**) HPLC analysis of serum from mice injected i.v. with ^124^I-PGN635 F(ab’)_2_ 48 h earlier. HPLC analysis showed an increase in the molecular weight of ^124^I-PGN635 F(ab’)_2_ with no evidence of lower molecular weight proteolytic fragments. **B**) Western blot analysis for β2GP1. PGN635 complexes were retrieved with protein A agarose from mouse serum 24 h after i.v. injection, and were probed with antibodies to β2GP1. The data show that PGN635 bound to circulating mouse β2GP1. Control antibody (Aurexis) collected from mouse serum 24 h after injection did not bind β2GP1.
(TIF)

Figure S2 **^125^I-PGN635 F(ab’)_2_ biodistribution at 24h.** Mice (n=3) bearing s.c. PC3-luc tumors were injected with 1.85 MBq (50 µg) of ^125^I-PGN635 F(ab’)_2_ or ^125^I-control F(ab’)_2_. Antibody distribution to the indicated organs was determined after 24 h by counting the radioactivity with a gamma counter. **A**) Biodistribution by percent injected dose per gram (%ID/g) of tissue. **B**) Biodistribution by percent injected dose per organ (%ID/organ). The % ID in the blood was calculated assuming a blood volume of 2.18ml/25 g body weight.
(TIF)

Figure S3 **Transverse images of subcutaneous PC3 tumors imaged with ^124^I-PGN635 F(ab’)_2_.** Transverse images also clearly show preferential labeling of the tumor by PET and relatively low uptake in normal tissues at 48 h post-injection. ^124^I-control F(ab’)_2_ did not label tumors.
(TIF)

Figure S4 **Sagittal images of orthotopic PC3-luc tumors imaged with ^124^I-PGN635 F(ab’)_2_.** Sagittal PET images clearly show preferential labeling of the orthotopic prostate tumor at 48 h post-injection. ^124^I-control F(ab’)_2_ did not label the tumors.
(TIF)

Figure S5 **Effects of CTx and xRT on the growth of subcutaneous PC3-luc tumors.** **A**) Growth curves for untreated PC3-luc tumors (n=3). Tumor-to-normal (T/N) ratios for ^124^I-PGN635 F(ab’)_2_ uptake were determined by PET imaging at 24 days after implantation (dashed arrow). Tumor volume increased by an average of 11.5-fold between day 21 and day 49. **B**) Growth curves for PC3-luc tumors treated with 10 mg/kg docetaxel (CTx) at 21 days after implantation (n=3). ^124^I-PGN635 F(ab’)_2_ PET imaging (dashed arrow) was performed 72 h after treatment. Tumor volume increased by an average of 2.4-fold 28 days after treatment. **C**) Growth curves for PC3-luc tumors irradiated with 15 Gy (xRT) at 21 days after implantation (n=3). Again, ^124^I-PGN635 F(ab’)_2_ PET imaging (dashed arrow) was performed 72 h after treatment. Tumor volume decreased by an average of 10% at 28 days after treatment.
(TIF)

Materials S1 (DOCX)
