# Supplementary figures and images for: Highly Specific PET Imaging of Prostate Tumors in Mice with an Iodine-124-Labeled Antibody Fragment That Targets Phosphatidylserine
Source: PLoS One. 2013 Dec 19;8(12):e84864. doi: 10.1371/journal.pone.0084864 (PMC3868598; doi:10.1371/journal.pone.0084864)

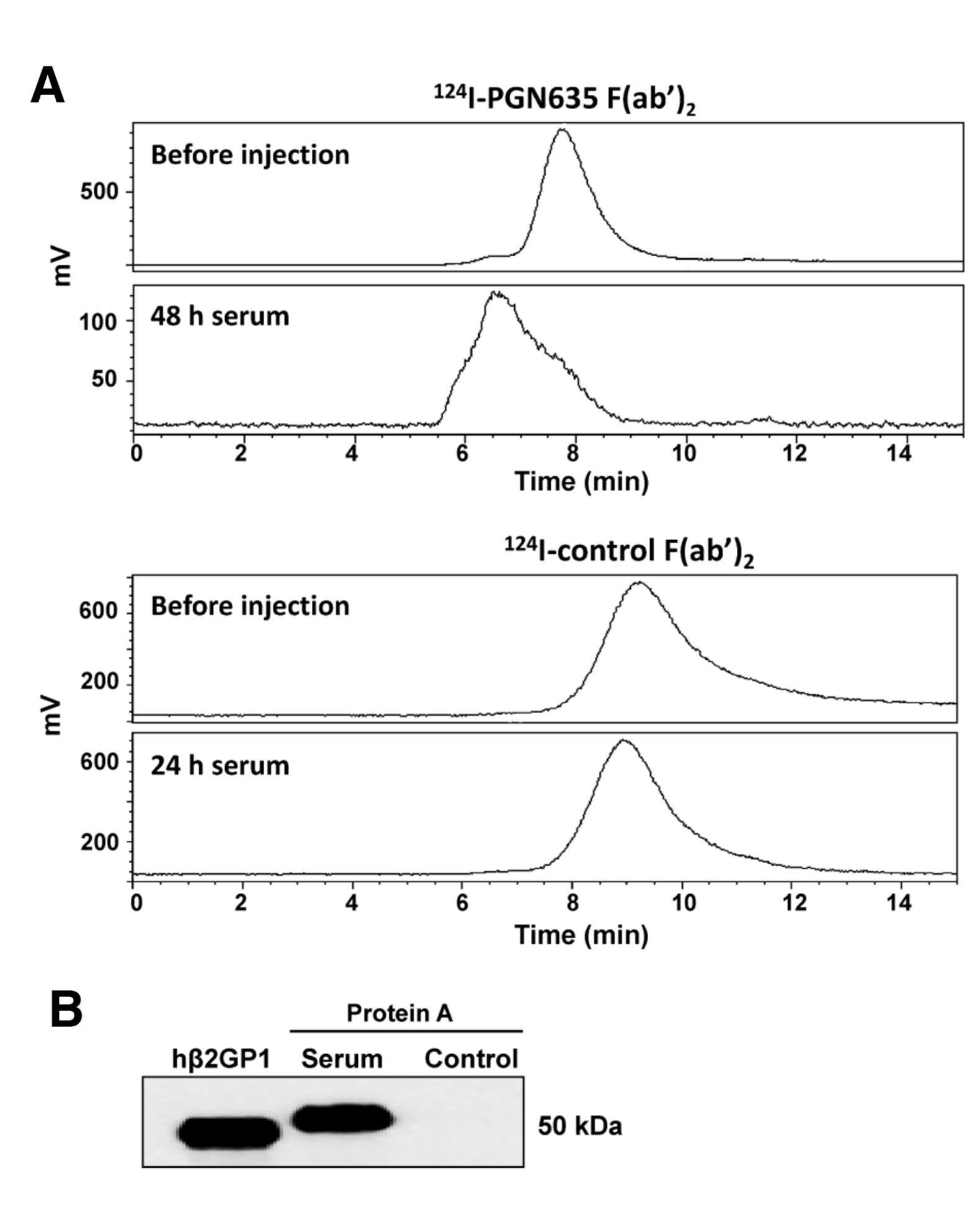

Supplement: Figure S1 — 124I-PGN635 F(ab’)2 is stable invivo and binds serum β2GP1. A) HPLC analysis of serum from mice injected i.v. with 124I-PGN635 F(ab’)2 48 h earlier. HPLC analysis showed an increase in the molecular weight of 124I-PGN635 F(ab’)2 with no evidence of lower molecular weight proteolytic fragments. B) Western blot analysis for β2GP1. PGN635 complexes were retrieved with protein A agarose from mouse serum 24 h after i.v. injection, and were probed with antibodies to β2GP1. The data show that PGN635 bound to circulating mouse β2GP1. Control antibody (Aurexis) collected from mouse serum 24 h after injection did not bind β2GP1. (TIF) [file pone.0084864.s001.tif]

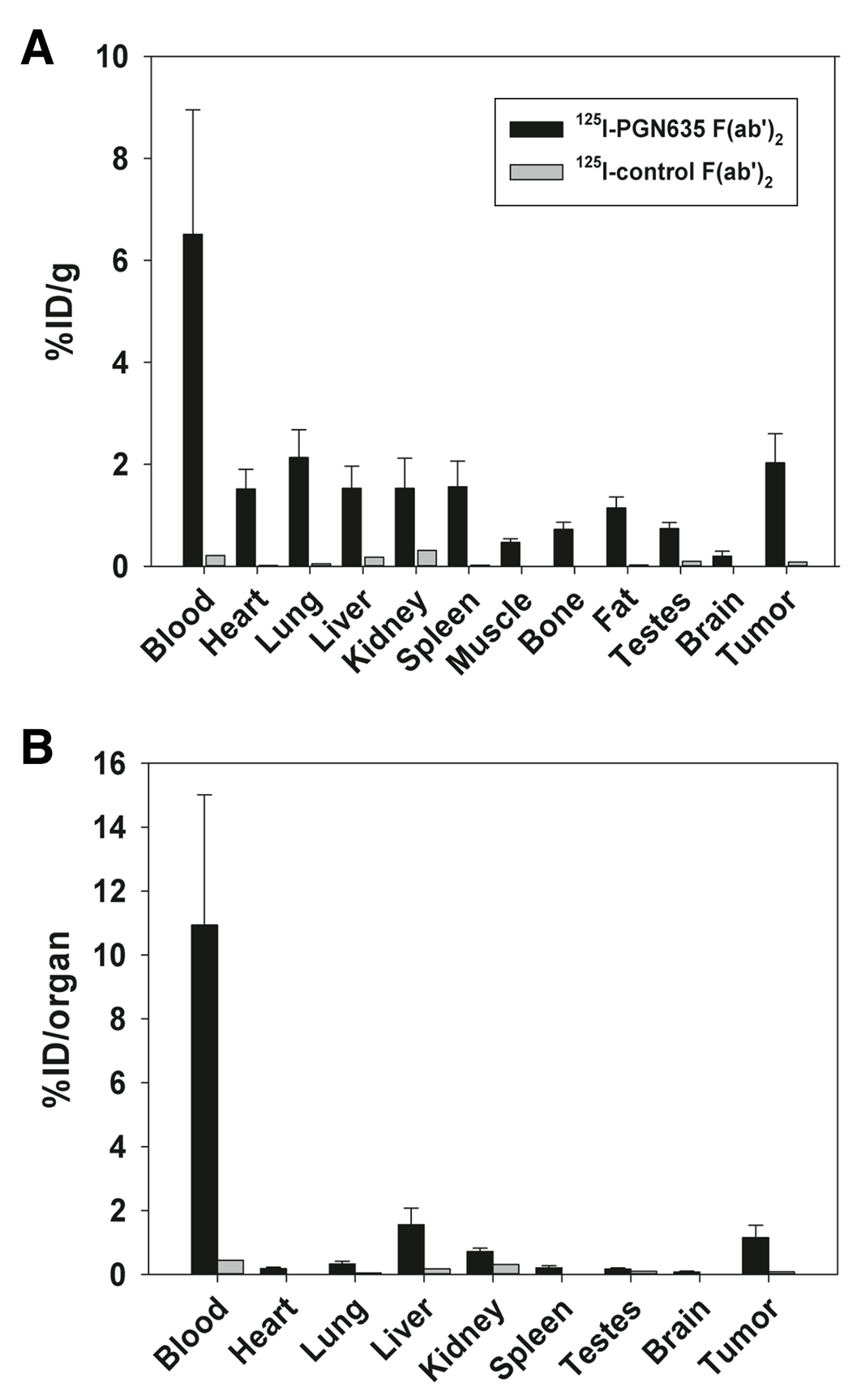

Supplement: Figure S2 — 125I-PGN635 F(ab’)2 biodistribution at 24h. Mice (n=3) bearing s.c. PC3-luc tumors were injected with 1.85 MBq (50 µg) of 125I-PGN635 F(ab’)2 or 125I-control F(ab’)2. Antibody distribution to the indicated organs was determined after 24 h by counting the radioactivity with a gamma counter. A) Biodistribution by percent injected dose per gram (%ID/g) of tissue. B) Biodistribution by percent injected dose per organ (%ID/organ). The % ID in the blood was calculated assuming a blood volume of 2.18ml/25 g body weight. (TIF) [file pone.0084864.s002.tif]

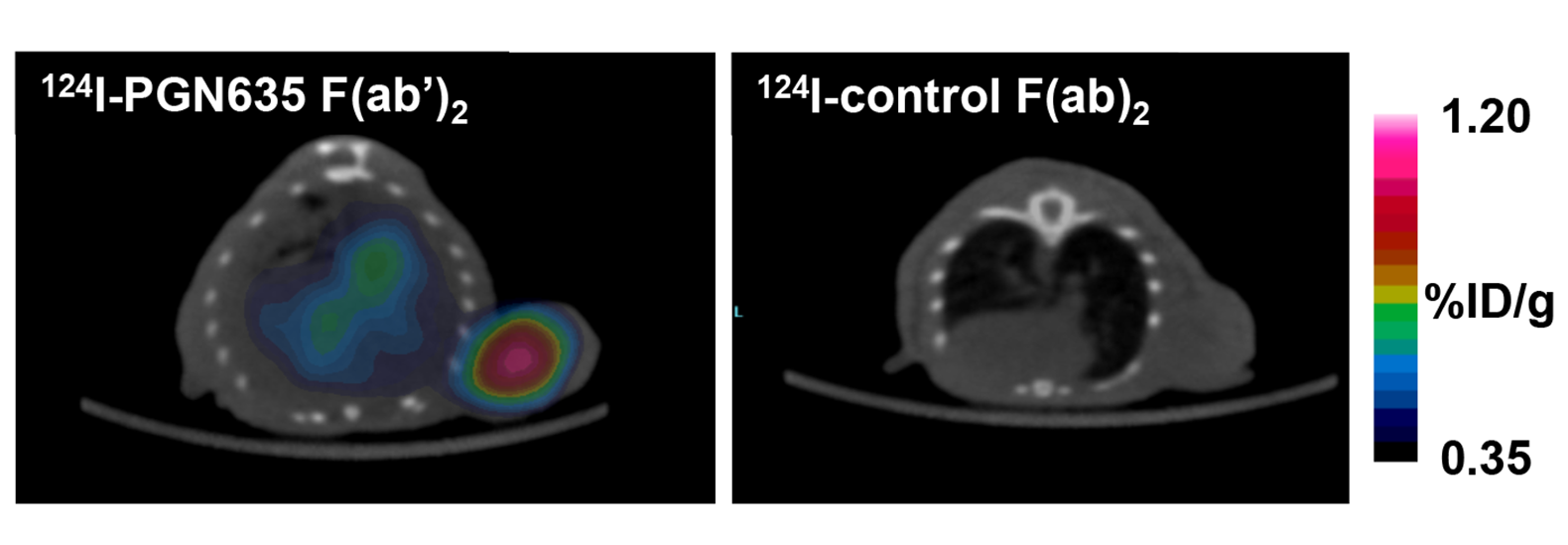

Supplement: Figure S3 — Transverse images of subcutaneous PC3 tumors imaged with 124I-PGN635 F(ab’)2. Transverse images also clearly show preferential labeling of the tumor by PET and relatively low uptake in normal tissues at 48 h post-injection. 124I-control F(ab’)2 did not label tumors. (TIF) [file pone.0084864.s003.tif]

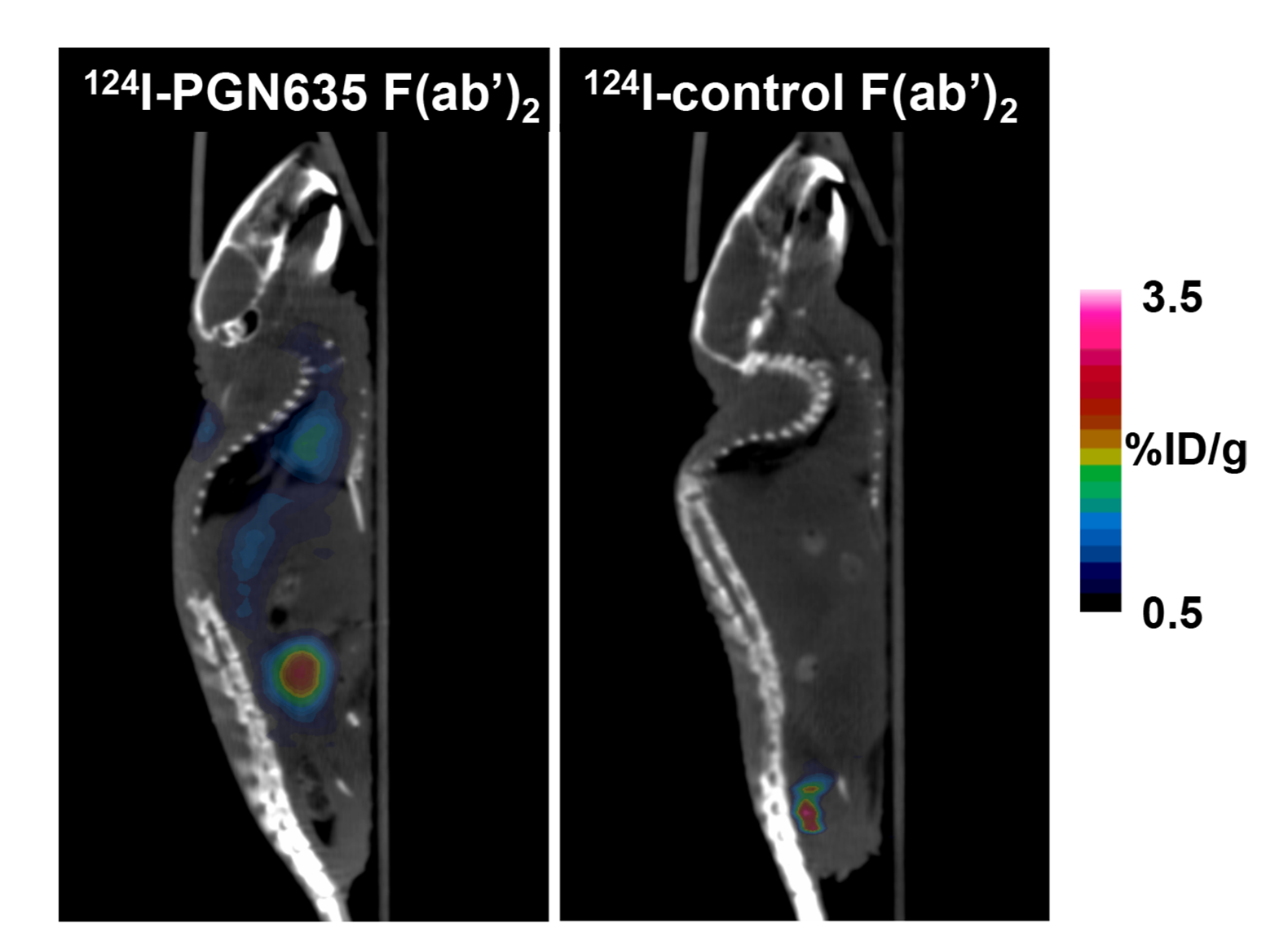

Supplement: Figure S4 — Sagittal images of orthotopic PC3-luc tumors imaged with 124I-PGN635 F(ab’)2. Sagittal PET images clearly show preferential labeling of the orthotopic prostate tumor at 48 h post-injection. 124I-control F(ab’)2 did not label the tumors. (TIF) [file pone.0084864.s004.tif]

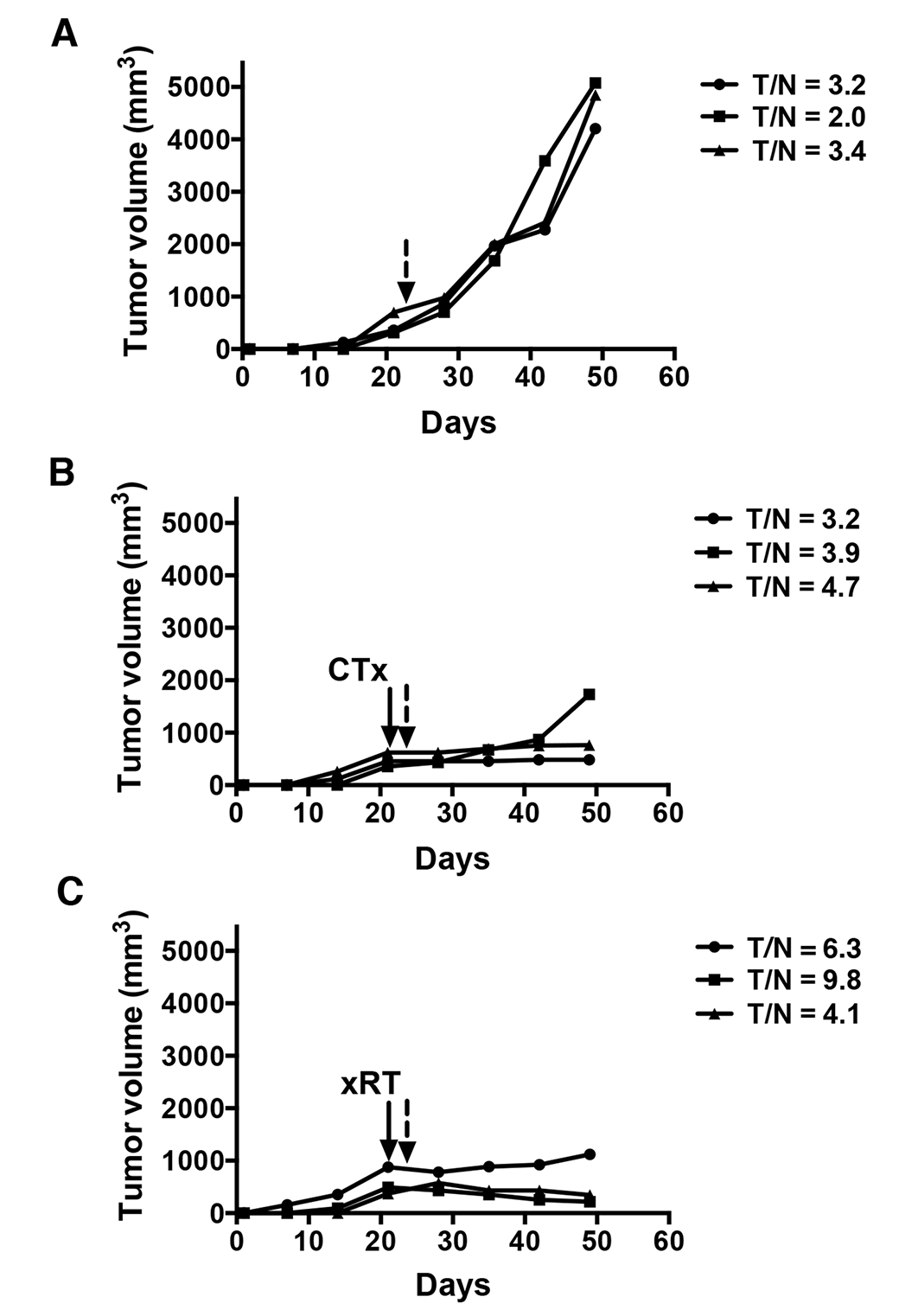

Supplement: Figure S5 — Effects of CTx and xRT on the growth of subcutaneous PC3-luc tumors. A) Growth curves for untreated PC3-luc tumors (n=3). Tumor-to-normal (T/N) ratios for 124I-PGN635 F(ab’)2 uptake were determined by PET imaging at 24 days after implantation (dashed arrow). Tumor volume increased by an average of 11.5-fold between day 21 and day 49. B) Growth curves for PC3-luc tumors treated with 10 mg/kg docetaxel (CTx) at 21 days after implantation (n=3). 124I-PGN635 F(ab’)2 PET imaging (dashed arrow) was performed 72 h after treatment. Tumor volume increased by an average of 2.4-fold 28 days after treatment. C) Growth curves for PC3-luc tumors irradiated with 15 Gy (xRT) at 21 days after implantation (n=3). Again, 124I-PGN635 F(ab’)2 PET imaging (dashed arrow) was performed 72 h after treatment. Tumor volume decreased by an average of 10% at 28 days after treatment. (TIF) [file pone.0084864.s005.tif]
