# Supplementary material for: Highly Specific PET Imaging of Prostate Tumors in Mice with an Iodine-124-Labeled Antibody Fragment That Targets Phosphatidylserine
Source: PLoS One. 2013 Dec 19;8(12):e84864. doi: 10.1371/journal.pone.0084864 (PMC3868598; doi:10.1371/journal.pone.0084864)
Supplement: Materials S1 — (DOCX) [file pone.0084864.s006.docx]

**SUPPLEMENTAL METHODS**

***In Vivo* Stability Test**. Non-tumor bearing mice were injected i.v. with either 50 µg, 1.85 MBq (50 µCi) or 50 µg, 3.7 MBq (100 µCi) doses of ^124^I-PGN635 F(ab’)_2_. Mice injected with 1.85 MBq were sacrificed at 1 h and 24 h after injection and mice injected with 3.7 MBq were sacrificed at 48 h after injection. Blood was collected and the serum was analyzed by HPLC with a Waters Biosuite 125 SEC column (300 x 7.8mm, 10 µm) and a Waters 600 Mulitsolvent Delivery System equipped with a Waters 2996 Photodiode Array (PDA) detector and in-line Shell Jr. 2000 radiodetector (Fredericksburg, VA). The mobile phase was PBS (pH = 7.2) at a 1.0 ml/min flow rate.

**Co-purification of PGN635 and β2GP1.** Mice were injected with 200 µg PGN635 IgG or Aurexis (control). After 24 h, the mice were sacrificed and blood collected. The serum was incubated with Protein A agarose beads for 1 h. The beads were washed with PBS and then boiled in Laemmli sample buffer without β-mercaptoethanol. Equal amounts of total protein were loaded for SDS-PAGE (4-15% Tris-HCl) and transferred to PVDF membranes. The membranes were blocked for 2 h in 5% non-fat milk/1% BSA, incubated for 1 h with polyclonal goat anti-β2GP1 antibody (1:5000 in 1% BSA) and then incubated with donkey anti-goat IgG antibody (1:10,000 in 1% BSA) for 30 min. The membranes were washed with 1% Tween-20 in PBS after each step. The membranes were developed with Amersham ECl western blotting detection reagents, exposed to radiographic film, and processed in a Kodak X-OMAT 1000A film processor (Eastman Kodak Co., Rochester, NY)
